# Efficacy of Human Papillomavirus Vaccines for Recalcitrant Anogenital and Oral Warts

**DOI:** 10.3390/jcm12237317

**Published:** 2023-11-26

**Authors:** Giulia Ciccarese, Astrid Herzum, Gaetano Serviddio, Corrado Occella, Aurora Parodi, Francesco Drago

**Affiliations:** 1 Unit of Dermatology, Department of Medical and Surgical Sciences, University of Foggia, Viale Pinto 1, 71122 Foggia, Italy; giulia.ciccarese@unifg.it; 2 Dermatology Unit, IRCCS Istituto Giannina Gaslini, Via G Gaslini 5, 16147 Genova, Italy; corradooccella@gaslini.org; 3 Liver Unit, C.U.R.E. (University Centre for Liver Disease Research and Treatment), Department of Medical and Surgical Sciences, University of Foggia, Viale Pinto 1, 71122 Foggia, Italy; gaetano.serviddio@unifg.it; 4 Section of Dermatology, Dipartimento di Scienze della Salute, University of Genoa, Ospedale-Policlinico San Martino, IRCCS, Largo R. Benzi, 10, 16132 Genova, Italy; aurora.parodi@unige.it (A.P.); frdrago@libero.it (F.D.)

**Keywords:** human papillomavirus infection, human papillomavirus vaccine, anogenital warts

## Abstract

Human papillomavirus (HPV) vaccines are preventive measures to decrease HPV infection rates. Knowledge of their efficacy as treatment options for anogenital warts (AGWs) and oral warts (OWs) is limited. To evaluate the efficacy of HPV vaccinations in recalcitrant AGWs and OWs (lesions persisting more than 6 months despite conventional treatments), we compared a group of patients treated with standard therapies plus an HPV vaccine with a group of patients treated with standard therapies only. The response to treatment (in terms of the number of lesions) in the two groups was compared. Data were analyzed with the χ2 test and *p* values < 0.05 were considered to be statistically significant. The study included 14 patients (group A = cases) who received 3 doses of an intramuscular HPV vaccine (Gardasil 4 or Gardasil 9) in addition to the standard treatments for AGWs and OWs, and 15 age- and sex-matched patients (group B = controls) with an analogous number of lesions to group A who received only standard therapies. After 12 months, 85% of patients of group A versus 33% of group B had positive clinical outcomes (0.004). Our findings suggest a possible therapeutic role of HPV vaccines in addition to standard treatments for AGWs/OWs. Preventive vaccines, blocking the viral entry through the induction of L1-specific antibodies, can prevent autologous reinfections (through auto-inoculation) and favor the elimination of the virus.

## 1. Introduction

Human papillomavirus (HPV) is the most common sexually transmitted infection (STI) worldwide, affecting sexually active individuals at least once in their lifetime [1,2,3]. Cervical cancer, the most prevalent cancer in females, is associated with persistent HPV infection in over 99% of cases, highlighting the importance of HPV infection prevention [2]. HPV is a small, double-stranded DNA virus, infecting the skin, epithelia of the oropharynx, cervix, vagina, vulva, penis, and anus [2,4]. It is well known that several HPVs harbor an oncogenic potential and the persistence of HPV in infected cutaneous and mucosal epithelia may induce carcinogenesis through the action of viral oncogenes [2]. HPV represents an extremely common ubiquitous pathogen and numerous HPV genotypes of high and low oncogenic potential normally reside on human skin and mucosae, mainly without causing symptomatic disease in immunocompetent subjects [5,6]. The difference in HPV’s ability to promote malignant transformations is the basis for the classification into low-risk (LR) and high-risk (HR) genotypes. Generally, in immunocompetent individuals, HPV infections are self-limiting as the host’s immune system will eventually clear them. However, in some individuals, especially if immunosuppressed, an HPV infection cannot be controlled by the immune system and viral persistence may lead to the excessive proliferation and instability of the genome of infected epithelial cells, symptomatic infection, and eventually carcinogenesis [7,8].

Globally, over 200 HPV genotypes exist with a relevant genetic variability [2,9]. Their classification is based on variability among the genetic sequences that encode for the major viral capsid protein L1 [2,9]. HPV specifically infects the basal epithelial cells of the mucosae and skin [2]. By differentiating, the basal epithelial cells allow sequential viral protein synthesis, thereby ensuring ultimate viral multiplication (latent or active) and the release of new virions. The infection may be clinically asymptomatic, remaining undiagnosed for a long time, or lead to a perceptible uncontrolled proliferation of infected epithelia, clinically manifesting with AGWs, OWs, and cutaneous warts as well as pre-malignant proliferations, including cervical intraepithelial neoplasia (CIN) and malignant proliferation [10,11,12]. Only part of an HPV-related lesion will progress to cancer, depending on multiple variables such as genetic, cultural, and geographic factors, which influence viral genome variability and individual intrinsic factors such as age, gender, general health state, and the infected body area [2,13,14,15,16].

Importantly, an HPV infection may impact the patient’s psychological, physical, and sexual health as well as the patient’s global quality of life (QOL), even leading to depressive symptoms and changes in sexual functioning [17].

The burden of HPV infections and HPV-related diseases has led to increasing efforts of the scientific community to develop efficacious strategies for infection prevention. Among these, HPV vaccines are being administered worldwide and have already proven effective in reducing HPV infection rates and HPV-related diseases [18,19]. In 2006, the first quadrivalent HPV vaccine (Gardasil^®^), containing protein antigens for HPV types 6, 11, 16, and 18, was commercialized in most industrialized countries. The bivalent vaccine (Cervarix^®^), licensed in 2007, targets HPV types 16 and 18. Lastly, the nonavalent HPV vaccine (Gardasil 9), licensed in 2014, targets HPV types 6, 11, 16, 18, 31, 33, 45, 52, and 58 [4,19]. These vaccines are composed of recombinant, non-infectious virus-like particles (VLP), antigenically identical to infection-causing HPV virions, and induce a humoral neutralizing immune response in the host that efficiently halts the viral uptake into basal epithelial cells [4]. Since the introduction of HPV vaccination programs worldwide in the late 2000s, a significant decrease in AGWs, the CIN2 detection rate, and HPV-associated cancers have been registered [20,21,22]. The HPV vaccination is currently recommended only as a preventive measure against a primary HPV infection and once HPV has been acquired, no recommendation is given by the current sexually transmitted infection guidelines to administer an HPV vaccination [23].

To date, knowledge of the possible therapeutic effect of the HPV vaccine on already-established HPV infections and HPV-related disease is limited [4,18]. Considering the significant psychological and social distress that these lesions often cause to patients [17] and their frequent recurrence after conventional therapies [19], a possible combined effect, both therapeutic and preventive, of an HPV vaccination, would have revolutionary potential.

In the present study, we aimed to evaluate the possible therapeutic efficacy of HPV vaccinations in the treatment of recalcitrant AGWs and OWs described as HPV-induced benign epithelial proliferations persisting for more than 6 months and not responding to at least one standard treatment.

## 2. Materials and Methods

We retrospectively studied patients who had visited the STI Outpatient Clinic (Dermatology Unit, Policlinico San Martino, Genoa, Italy) between January 2015 and July 2019 for recalcitrant AGWs/OWs. Patients who received 3 doses of an intramuscular HPV vaccine in addition to and simultaneously with standard treatments for AGWs and OWs were included in the study as the cases (group A). Controls (group B) were selected from patients affected by recalcitrant AGWs/OWs who received only standard therapies, who were matched for age and sex, and who had clinical characteristics comparable with group A (analogous comorbidities and number of lesions). Demographic and clinical features were collected. The response to treatment was clinically evaluated at 12 months, based on the number of AGWs/OWs. The response was considered to be complete if there was a complete clearance of lesions, partial if there was a decrease > 50% in the number of lesions, and absent if there was a decrease < 50% in the number of lesions. The follow-up time was 12 months for all patients. Data were analyzed using the χ2 test and *p* values < 0.05 were considered to be statistically significant.

## 3. Results

The study included 14 patients (11 males and 3 females; average age of 39 years) in group A (cases) and 15 patients (10 males and 5 females; average age of 39 years) in group B (controls).

The clinical features of each patient are described in Table 1.

Clinically, the body area most frequently affected by warts was the genital area, followed by the perianal skin. The number of warts at the first visit ranged from 1 to 15 (average number: 7 in group A; 5 in group B) and their duration prior to the visit ranged from 6 months to 36 years (average duration: 5 years in group A; 2 years in group B). The most commonly used standard treatment was cryotherapy, followed by imiquimod 5% cream (Table 2).

Comorbidities affecting the immune system were present in 3 patients of group A (2 human immunodeficiency virus (HIV)-infected patients, both on antiretroviral therapy for 2 and 4 years; 1 autologous bone marrow transplant patient for Hodgkin’s lymphoma) and in 2 patients of group B (both having received autologous bone marrow transplantations for Hodgkin’s lymphoma less than one year before the first visit to our outpatient clinic). In group A, all the patients received the 9vHPV vaccine at the time of the study; 3 of them had also received the 4vHPV quadrivalent vaccine several years earlier. The difference in the clinical outcome between patients who received only the 9vHPV vaccine and patients who had previously received the 4vHPV vaccine was not statistically significant (*p* > 0.05).

Regarding the factors contributing to vaccine efficiency in group A, we found that an age < 45 years, a number of lesions less than 10, a duration of the lesions ≤ 2 years, and an absence of comorbidities affecting the immune system (as HIV infection and malignancies) were correlated with a better clinical outcome (a higher rate of complete/partial response to treatment in patients with these features compared with patients without), although the difference was not statistically significant (*p* > 0.05).

The response to treatment was clinically evaluated at 12 months. In group A, 9 patients showed a complete response (64%), 3 showed a partial response (21%), and 2 showed no response (15%). In group B, 4 patients showed complete responses (27%) and 9 responses were absent (60%). Taking the complete and partial responses to treatments together, 12 patients of group A (85%) versus 6 of group B (40%) had a positive clinical outcome (Table 3). The difference in the clinical outcome between group A and group B was statistically significant (*p* = 0.01).

## 4. Discussion

Nowadays, HPV vaccines are successfully administered as a primary antiviral prophylaxis in presumed HPV-naïve patients (adolescents at age 11 or 12 years) and in adults aged >26 years not previously vaccinated. HPV vaccines can be administered regardless of a history of AGWs, an abnormal Pap test or HPV test, or anogenital precancerous conditions. A 2-dose vaccine schedule (at 0- and 6–12-month intervals) is recommended for people who initiate vaccinations before their 15th birthday. A 3-dose vaccine schedule (at 0-, 1–2-, and 6-month intervals) is recommended for immunocompromised people, regardless of the age of initiation [23].

Recent studies have assessed the effectiveness of HPV vaccines in reducing the prevalence and mortality of HPV-related diseases among men who have sex with men (MSM) [24] and women with prior cervical disease [25].

Little is known about the possible role of HPV vaccines as therapeutic agents in already-infected individuals with ongoing HPV-related cutaneous and/or mucosal conditions [18,26].

Numerous conventional and alternative treatments exist for AGWs, including topical agents and physically destructive procedures [18,23]. There is no definitive evidence to indicate that any recommended treatment is superior to another, and no single treatment is ideal for all patients. The treatment choice should be guided by the wart size, number, anatomic site, patient preference, cost of treatment, convenience, adverse effects, and provider experience [23].

In the context of the many available therapies for AGWs and OWs, a possible dual role of HPV vaccines, both preventive and therapeutic, would be of revolutionary clinical relevance and deserves in-depth investigations and clinical research.

However, to date, only a few studies and sporadic case reports have focused on the possible therapeutic role of HPV vaccines (both the 2vHPVvaccine and 4vHPV vaccine); these obtained partly contradictory results [27,28,29,30,31,32,33,34,35].

The therapeutic efficacy of the last commercialized 9vHPV vaccine on AGWs was assessed only recently in three small case series [32] and in two case reports [33,34] as well as in the present report.

Bossart et al. reported 100% remission (40% complete; 60% partial) of recalcitrant AGWs in five male patients, 21 to 58 years old, after three doses of Gardasil 9 [33]. Similarly, Dianzani et al. recently reported three cases of male patients (22, 42, and 65 years old) with recalcitrant AGWs and plantar warts who obtained 100% complete remission after the administration of three doses of Gardasil-9^®^ [32].

Interestingly, in the present report, we observed a good response to HPV vaccines in both immunocompetent and immunocompromised patients (people living with HIV and patients who had recently received a bone marrow transplant).

Moreover, our study is the first to show the encouraging therapeutic results of the 9vHPV vaccine in patients with OWs. Cyrus et al. reported the complete remission of HPV-32-related oral papillomas in a 60-year-old immunocompetent man after three doses of the 4vHPV vaccine [36].

The exact immunologic phenomena underlying the therapeutic effect of HPV vaccines have not yet been elucidated. Whilst the preventive efficacy of commercially available HPV vaccines is based on the induction of a humoral immune response, it has been postulated that the regression of already-existing anal, vulvar, and cervical lesions is mainly associated with the activation of cellular, rather than humoral, immunity [37]. Originally, HPV vaccines were designed to generate neutralizing IgG antibodies directed against the major viral capsid protein L1, thereby preventing the infection by blocking the viral entry into the hosts’ epithelial cells [38]. Notably, the significant homology of the L1 capsid protein between various HPV types may result in cross-protection against different HPV types, even if not targeted by the vaccine [39,40].

Moreover, HPV vaccines have been associated with increased levels of cytokines (IL-2, IL-6, IL-1α, and IL-1β) and with the induction of IFN-γ-producing CD4+ and CD8+ T-lymphocytes [41], cytotoxic cells that clear viral infections. Similar changes in the local cytokine microenvironment may have a role in contrasting HPV-induced proliferation and in the clearance of HPV-persistent infections [42].

Several trials have indicated that the Bacillus Calmette–Guérin (BCG) vaccination has a protective and therapeutic efficacy against cutaneous and genital warts. Its efficacy is possibly due to a trained immunity’s cross-protection against other bacterial or viral infections through the efficient activation of the immune system, especially innate immune cells. Trained innate immunity is indeed characterized by providing cross-protection against infectious diseases following stimulation derived from bacteria, fungi, viruses, and parasites. This protective effect defends against a certain range of unrelated pathogens [43].

Similarly, it could be speculated that through the mechanism of trained immunity, HPV vaccines may efficiently activate innate immune cells against an already-present HPV infection.

HPV vaccines might be especially effective for AGWs as the DNA of LR HPVs is usually not integrated into the host genome and viral particles are produced and released from the granular layer of the epithelium at every viral replication [44]. HPV vaccines can prevent viral reinfections (that can occur through auto-inoculation) by blocking the viral entry into host cells, favoring viral elimination through L1-specific antibodies over time.

Conversely, in high-grade epithelial lesions, the HPV genome is often integrated into the host genome and infected cells neither express L1 nor produce viral particles. Therefore, as infected cells cannot be recognized by vaccine-induced antibodies, the HPV vaccination is ineffective in disease control [38].

## 5. Conclusions

In conclusion, our findings suggest that the addition of HPV vaccines to the standard treatments for AGWs and OWs leads to positive clinical outcomes. HPV vaccine administration together with conventional treatments might reduce the use of alternative and off-label treatments such as podophyllin resin, intralesional interferon, photodynamic therapy, and topical cidofovir that might be aggressive and associated with side-effects [23]. Although HPV vaccines are intended to produce neutralizing antibodies to avoid infection and reinfection, their ability to induce cell-mediated immunological responses, confirmed in previous studies [45,46], may be responsible for the regression of genital and anal lesions.

A limitation of our study was the small sample size. Larger randomized and controlled trials are needed to confirm the results of our study and to assess the mechanisms underlying a possible therapeutic effect of HPV vaccinations.

## Figures and Tables

**Table 1:** Clinical features of each patient in group A (cases) and in group B (controls).

Patient No.	Sex	Age at First Visit in Our STI Center	Site of the Warts	No. of Lesions	Duration (Years)	Comorbidities Affecting the Immune System	Previous Treatments	Current Standard Treatment	9vHPV Vaccination (yes/not)	Previous 4vHPV Vaccination	Response to Treatment
**cases (vaccinated patients)**											
1	F	25	genitalia	15	2	none	laser, imiquimod, pophillotoxin	cryotherapy, nitric zinc complex, photodinamic therapy	yes	no	partial
2	M	35	genitalia	11	5	none	cryotherapy, imiquimod	cryotherapy	yes	no	complete
3	M	29	genitalia	7	0.5	none	imiquimod	nitric zinc complex	yes	no	complete
4	M	46	genitalia	1	2	none	imiquimod	cryotherapy	yes	no	complete
5	M	30	genitalia	2	7	none	cryotherapy, imiquimod	cryotherapy	yes	yes (2 years earlier)	complete
6	M	64	tongue	3	2	HIV infection	surgery	cryotherapy	yes	no	complete
7	M	60	perianal skin	2	2.5	HIV infection	cryotherapy	cryotherapy	yes	no	no
8	M	56	genitalia	2	1	none	cryotherapy	nitric zinc complex	yes	no	complete
9	M	44	perianal skin	6	2	none	imiquimod	imiquimod	yes	no	complete
10	F	17	perianal skin	12	6	none	cryotherapy, nitric zinc complex, photodinamic therapy	cryotherapy	yes	yes (5 years earlier)	complete
11	M	22	genitalia	12	2	Hodgkin lymphoma (bone marrow transplant 4 months before)	cryotherapy, photodinamic therapy	cryotherapy	yes	no	partial
12	F	51	tongue and cheek mucosa	13	36	none	intralesional interferon	cryotherapy	yes	yes (2 years earlier)	no
13	M	29	genitalia	13	1	none	cryotherapy	imiquimod	yes	no	partial
14	M	23	genitalia	5	3	none	cryotherapy	cryotherapy	yes	no	complete
**controls (not vaccinated)**											
1	F	35	genitalia and perianal skin	3	8	none	surgery, salicylic acid, sinecatechins	nitric zinc complex	no		no
2	M	28	genitalia	10	5	none	cryotherapy, laser	imiquimod	no		complete
3	M	24	genitalia	3	1	none	imiquimod	cryotherapy, nitric zinc complex	no		partial
4	M	43	genitalia	10	2	none	laser, surgery	cryotherapy	no		complete
5	M	57	genitalia	5	1	none	cryotherapy	nitric zinc complex	no		complete
6	M	63	genitalia	2	0.5	gastric lymphoma	surgery	cryotherapy	no		partial
7	M	59	perianal skin	4	0.5	Hodgkin lymphoma (bone marrow transplant 6 months before)	imiquimod	nitric zinc complex	no		no
8	M	45	genitalia	2	1	none	surgery	nitric zinc complex	no		no
9	F	27	genitalia	9	0.5	none	none	sinecatechins, imiquimod	no		no
10	M	28	genitalia	7	0.5	Hodgkin lymphoma (bone marrow transplant 6 months before)	cryotherapy	cryotherapy, imiquimid	no		no
11	F	40	genitalia	10	2	none	surgery	cryotherapy, imiquimod	no		no
12	M	54	genitalia	1	0.5	none	imiquimod	cryotherapy	no		no
13	M	26	genitalia	6	0.5	none	cryotherapy	nitric zinc complex	no		complete
14	F	40	genitalia	7	1	none	imiquimod	cryotherapy	no		no
15	F	58	genitalia	4	2	none	surgery	cryotherapy	no		no

**Table 2:** Demographic and clinical features of the studied patients.

Variables	Group A (Vaccine + Standard Therapies)	Group B (Standard Therapies)
No. of patients	14	15
Males	11	10
Females	3	5
Age (years)	Range: 17–64 (mean age: 39)	Range: 24–63 (mean age: 39)
No. of lesions	1–15 (mean: 7)	1–10 (mean: 5)
Duration (years)	0.5–36 (mean: 5)	0.5–8 (mean: 2)
**Site of Lesions**		
Genital area	9	14
Perianal area	3	2
Oral mucosa	2	0
**Current Standard Therapies**		
Cryotherapy	9	8
Nitrizinc complex	3	6
Imiquimod 5% cream	2	3
Photodynamic therapy	1	0
**Comorbidities Affecting the Immune System**		
Human immunodeficiency virus infection	2	0
Hodgkin’s lymphoma (post-bone marrow transplantation)	1	2

**Table 3:** Response to treatments of the two study groups.

	Group A	Group B
**Response at 12 Months**		
No response	2 (15%)	9 (60%)
Partial response	3 (21%)	2 (13%)
Complete response	9 (64%)	4 (27%)

## Data Availability

Details on the data of these study are not made public for privacy reasons; however, they are available on reasonable request to the corresponding author.
